# Redefining Clinical Trials on Regenerative Therapies to Target Responsiveness in Temporomandibular Joint Osteoarthritis Treatment

**DOI:** 10.1111/joor.14012

**Published:** 2025-05-19

**Authors:** Ricardo de Souza Tesch, Rosana Bizon Vieira Carias, Thayanne Brasil Barbosa Calcia, Priscila Grion de Miranda Borchio, Esther Rieko Takamori

**Affiliations:** ^1^ Petrópolis Medical School/UNIFASE/Regenerative Medicine Laboratory Petrópolis Brazil; ^2^ Petrópolis Medical School/UNIFASE/Clinical Research Center Petrópolis Brazil

**Keywords:** cell‐based therapies, clinical trials, osteoarthritis, temporomandibular joint

## Abstract

**Background:**

Temporomandibular joint osteoarthritis (TMJ‐OA) presents a significant clinical challenge, marked by limited therapeutic options and variable patient responses. While regenerative therapies show promising results, conventional clinical trial methodologies often fail to demonstrate a significant clinical difference, largely due to response variabilities, which is critical in complex conditions like TMJ‐OA. As such, methodologies traditionally used in clinical trials for regenerative therapies targeting TMJ‐OA may need to be restructured to better identify and accommodate the distinctive profiles of responders and non‐responders.

**Objective:**

This paper proposes a revised clinical trial framework that focuses on differentiating responders from non‐responders within treatment groups, rather than relying solely on traditional control groups.

**Methods:**

Analysing non‐responders provides valuable insights for therapeutic individualisation and optimises patient selection, as understanding predictive factors can lead to improved outcomes. We present recommendations that integrate imaging diagnostics, chronic pain characteristics, and psychosocial assessments, offering a novel approach to enhance efficacy of TMJ‐OA treatment.

**Conclusion:**

This work advocates for a re‐evaluation of standard clinical trial designs to support more personalised and effective strategies for the management of TMJ‐OA.

## Graphical abstract

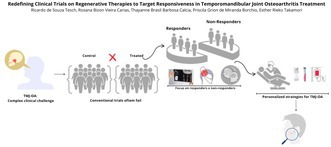

## 1 Introduction

Temporomandibular joint osteoarthritis (TMJ‐OA) is a degenerative and inflammatory joint disorder that affects the structural components of the TMJ, including articular cartilage, subchondral bone and associated soft tissues [1]. Its prevalence is higher among older females, and it is often associated with chronic pain, mandibular functional impairment and reduced quality of life. In advanced stages, significant disability and emotional distress are commonly observed [2]. Understanding the interplay between TMJ‐OA, aging and quality of life is essential for the development of effective treatment strategies, especially for older individuals who may have already undergone multiples therapeutic options.

Conventional treatments for TMJ‐OA, including intra‐oral appliances, pharmacotherapy, physical therapy, and surgical interventions, often yield inconsistent results and may be ineffective in cases of advanced degeneration or complex pathophysiology. Consequently, there has been growing global interest in biological therapies for TMJ‐OA. Regenerative therapies have a history spanning over two centuries, with various cell types and their derivatives being employed to treat a broad spectrum of diseases [3]. In the context of TMJ‐OA, their efficacy appears to be influenced by the severity of the condition. For example, intra‐articular injections of injectable platelet‐rich fibrin (I‐PRF) injections have demonstrated significant pain reduction in many patients with painful TMJ‐OA, with treatment success seemingly dependent on disease stage. In a non‐controlled clinical trial, 69% of patients receiving TMJ I‐PRF injections showed significant pain relief at eight weeks, which effects sustained at 3, 6, and 12 months, classifying them as responders. The remaining 31% showed no improvement and were classified as non‐responders. Surprisingly, the most favorable responses to I‐PRF injections were observed in individuals with more advanced stages of the disease [4].

Along with I‐PRF, PRP and other blood derivatives ‐ which collectively represent a source of growth factors and may also function as scaffolds ‐ cell‐based therapies constitute a key component of the regenerative medicine tripod and offer a promising alternative to surgery for treating severe TMJ‐OA. Emerging cell‐based therapies hold the potential to halt or even reverse the progression of TMJ‐OA; however, their effectiveness has been shown to vary significantly among patients. Available data on this topic indicate a high percentage of non‐responders in clinical trials involving cell‐based therapies, particularly those not targeting the haematopoietic system. In these trials, approximately 50% of patients did not respond to therapies such as mesenchymal stem cells within the first 1–3 months. Nevertheless, among the initial responders, most experienced sustained pain relief for up to three years [5]. Given that these therapies are autologous and have been demonstrated to be safe [6], regenerative treatments should be directed toward patients identified as having a greater likelihood of responding.

Traditional clinical trial designs, which compare intervention and control groups, may not adequately capture individual variability in response, particularly in a heterogeneous condition such as TMJ‐OA. Diverse symptom profiles and pathophysiological factors can substantially influence clnical outcomes. Methodologies aimed at for distinguishing responders from non‐responders represent a crucial component of medical research and healthcare. They seek to determine which patients benefit from a given treatment or intervention and which do not. This process is essential for optimising patient care, refining treatment strategies, and advancing medical knowledge. Thus, identifying responders and non‐responders within the treatment cohort can yield valuable insights into the predictors of therapeutic success and failure, addressing a critical gap in personalised approaches to TMJ‐OA management. In this context, we propose a revision of the testing framework for regenerative therapies in TMJ‐OA, incorporating methodological refinements aimed at establishing responsiveness profiles.

## 2 Methods

Clinical trials evaluating the effects of cell‐based therapies in TMJ‐OA can be significantly improved mostly by focusing on two key variables: enhanced patient selection and more robust data analysis. Thus, it is of essential to discuss strategies aimed at strengthening these domains (Table 1). Furthermore, quality control remains a critical concern in cell‐based therapy studies and should also be included in the discussion.

**TABLE 1:** Main suggestions for cell‐based therapies trial refinement.

**Inclusion criteria for enhanced patient stratification**
Imaging diagnostics Pain domains Psychosocial assessment
**Data analysis enrichment**
Emphasising responder and non‐responder profiling in trials design toward: Quantitative imaging Pain and function metrics Psychological and qualitative sensory measures
**Standardisation of advanced cell‐based therapy and quality control**

### 2.1 Inclusion Criteria for Enhanced Patient Stratification

Firstly, it is important to emphasise that appropriate patient selection is a crucial factor when investigating regenerative therapies for TMJ‐OA. Recommendations for intra‐articular injection therapies vary across clinical guidelines, and a consensus on their overall efficacy OA treatment has yet to be reached. Nonetheless, predictive factors have been identified to help distinguish patients who are more likely to benefit from such therapies. For instance, evidence indicates that the initial response to a single hyaluronic injection is a strong predictor of long‐term outcomes, particularly in patients with mild‐to‐moderate knee osteoarthritis and less severe structural damage [7, 8]. In contrast, non‐responsive patients are more likely to proceed to arthroplasty [9]. Therefore, careful patient selection may enhance the outcomes of intra‐articular injections. To improve precision in this process, we recommend using multidimensional inclusion criteria that incorporate structural imaging characteristics, pain chronicity, and psychological profiling.

#### 2.1.1 Imaging Features

Advanced imaging techniques, particularly magnetic resonance imaging (MRI), should be prioritised in the assessment of TMJ‐OA to evaluate joint cartilage, bone integrity, synovial inflammation, and disc morphology. Computed tomography (CT) may serve as complementary tool to MRI for the evaluation of bony changes when necessary. MRI‐based structural phenotypes in TMJ‐OA underscore the complexity of the condition, and the identification of distinct phenotypes offers valuable insights for personalised therapeutic interventions. This approach, which has been successfully applied to patient stratification in knee osteoarthritis trials, may likewise serve as a key pathway for the development of more effective treatments [10]. Tools such as the ROAMES‐TMJ (Rapid OsteoArthritis MRI Eligibility Score for TMJ) are designed to classify patients into specific structural phenotypes, thereby facilitating tailored treatment strategies and reinforcing the importance of individualised care [11].

#### 2.1.2 Pain Domains

Given the impact of pain chronicity on treatment outcomes, documenting pain duration and sensory patterns is essential. Although these factors may not directly correlate with the stages of joint degeneration [12, 13], they offer predictive insights into atypical patterns of treatment responsiveness. Patient‐based studies support the notion that pain‐sensitive patients—those exhibiting lower pressure pain thresholds (PPT), as measured by Quantitative Sensory Testing (QST), compared to healthy controls, but without psychological distress—constitute a distinct subgroup when compared to individuals within the adaptive cluster [14]. Despite their hypersensitivity, these patients do not exhibit psychological comorbidities, but rather demonstrate heightened orofacial and cervical hypersensitivity, suggesting significant local or segmental sensitisation [15]. Using PPT as a marker of pain sensitivity for clustering ‐ rather than focusing solely on anatomical pain localization ‐ may provide a more effective method for identifying individuals at risk of developing persistent chronic pain, even in cases where tissue regeneration is achieved. These variations in pain assessments suggest that distinct mechanisms may underlie pain among patients across different clusters. Consequently, personalised treatment strategies may be required to optimise care, particularly for individuals diagnosed with TMJ‐OA.

#### 2.1.3 Psychosocial Assessment

Psychological factors, including depression, anxiety and general somatic complaints, significantly influence pain perception and treatment response in chronic conditions. Insights from the OPPERA study (Orofacial Pain: Prospective Evaluation and Risk Assessment) underscore the relevance of these factors in pain management [16]. Patients classified within the Global Symptoms cluster exhibit not only lower regional pressure pain thresholds but also a combination of heightened pain sensitivity and comorbid psychological symptoms [14]. As previously reported, characteristics such as helplessness may be intrinsically related to pain severity in knee osteoarthritis [17]. This complexity in the biopsychosocial underpinnings of pain may therefore be associated with a poorer prognosis in patients with TMJ‐OA. Incorporating psychosocial evaluations into the clinical framework can facilitate the identiication of patients who could benefit from adjunctive psychological support. Such integrated approaches, when combined with cell‐based therapies, have the potential to optimise treatment outcomes for TMJ‐OA.

### 2.2 Enriching Data Analysis

#### 2.2.1 Evidences From Advanced Cell‐Based Therapies RCT for TMJ‐OA

To the best of our knowledge, only three published phase II clinical trials have investigated cell‐based therapies for TMJ‐OA management [18, 19, 20] (Table 2). Riu et al. (2019) [18] conducted a study involving 30 patients with unilateral degenerative temporomandibular joint disorders and internal derangement, dividing them into two groups: one receiving TMJ arthrocentesis followed by hyaluronic acid (HA) injection, and the other receiving arthrocentesis followed by an intra‐articular injection of bone marrow mononuclear cells (BMNc). Both groups experienced significant clinical improvement over 12 months. However, the BMNc group exhibited superior outcomes, including greater pain relief, improved interincisal mouth opening at 6 and 12 months, and enhanced chewing efficiency at 12 months.

**TABLE 2:** Main characteristics of studies that addressed cell‐based therapies in TMJ degenerative joint disease.

Reference	Patients' features	Inclusion criteria	Experimental groups	Outcomes and timepoint assessments	Main results	Strengths	Limitations
De Riu et al. (2019) [18]	30 patients with unilateral degenerative temporomandibular joint disorders and internal derangement	Age between 18 to 70 years old. Chronic temporomandibular arthritic disorder with history of pain and joint noises for at least 1 year. Spontaneous and evoked TMJ pain (with mandibular movements and direct TMJ compression). Wilkes stages III or IV (internal derangement). Joint noises, such as crepitation and clicking. Limited mouth opening Magnetic resonance imaging evidence of cartilage surface defects. Already treated with conservative methods for at least 3 months without satisfactory benefit.	RCT with two groups: Arthrocentesis followed by an intra‐articular injection in TMJ superior compartment of: Hyaluronic acid (HA) (control) or Bone marrow mononuclear cells (BMNc) (experimental)	Pain at rest and during movement (VAS), joint noises (VAS), chewing efficiency (VAS), and maximum interincisal opening (MIO), with MRI scans for cartilage assessment. 7 days, 1 month, 6 months, and 12 months after the procedure	Both groups showed significant clinical improvement over 12 months. However, the BMNc group demonstrated superior pain relief at 6 and 12 months, better chewing efficiency after 12 months, and greater MIO gains at both 6 and 12 months. No significant differences in joint noises or MRI evidence of cartilage regeneration were found.	Proper image analysis made in inclusion and cartilage assessment post therapy.	Lacks analysis of biomarkers or other characteristics that might distinguish responders from non‐responders. Unidimensional approach of pain measurement. Lack of psychosocial assessment.
Sembronio et al. (2021) [19]	40 patients with unilateral or bilateral internal derangement and TMJ osteoarthritis	TMJ internal derangement and osteoarthritis assessed by clinical examination and magnetic resonance imaging. Presence of TMJ‐related symptoms including at least limited mouth opening and joint pain. Previously failed conservative treatment. Age superior to 16 years. No previous TMJ surgical procedures.	RCT with two groups: Arthrocentesis followed by an intra‐articular injection in TMJ superior compartment of: Hyaluronic acid (HA) (control) or Microfragmented adipose tissue (experimental)	Pain (VAS) and MIO Follow‐up period of 10 days, 1 month and 6 months.	Both groups showed significant post‐treatment improvements in pain and mouth opening (*p* = 0.001). However, the success rates, defined as a mouth opening ≥ 35 mm and VAS ≤ 2, were achieved in 50% of the control group and 85% of the experimental group, with a significant difference favouring the experimental group (*p* = 0.018).	First study on the therapeutic effect of injection of microfragmented adipose tissue for TMJ osteoarthritis	Lacks analysis of biomarkers or other characteristics that might distinguish responders from non‐responders. Unidimensional approach of pain measurement. Lack of psychosocial assessment. Lack of image assessment in follow‐up. Shorter follow‐up time (six months).
Tesch et al., 2024 [20]	10 patients with severe degenerative joint disease	Age superior to 18. Dentofacial deformities related to severe degenerative disease of the TMJ, irrespective of the presence or absence of concomitant arthralgia. Surgical indication for orthognathic surgery to correct dentofacial deformity.	Open‐label I/IIa study enrolling patients submitted to arthrocentesis followed by single intra‐articular injection of autologous chondroprogenitor cells, in both TMJs.	Pain (Von Korff scale) Mandibular function (range of motion unassisted and assisted) Condylar structural changes in computerised tomography Follow‐up period at 7 and 15 days, 1, 3, 6, and 12 months	Autologous chondroprogenitors effectively reduced arthralgia, improved mandibular function, and either stabilised condylar volume or promoted tissue regeneration in specific cases. All patients experienced improved pain‐free and assisted mouth opening, with most condyles showing stabilisation of degenerative changes without significant further loss of volume	Applied DC/TMD to follow‐up appointments, addressing pain and mandibular function. Assessment of structural changes in TMJ. Presence of safety analysis.	Not possible to realise responsiveness analysis due to the small number of patients. Lack of control group.

Sembronio et al. (2021) [19] enrolled 40 patients with TMJ internal derangement and osteoarthritis, dividing them into two groups to compare standard TMJ treatment with HA injections (control) to an innovative approach using microfragmented adipose tissue injections, both administered post‐arthrocentesis. Both groups achieved significant post‐treatment improvements in pain and mouth opening (*p* = 0.001). However, success rates—defined as a mouth opening ≥ 35 mm and VAS ≤ 2—were observed in 50% of the control group and 85% of the experimental group, with a significant advantage favouring the experimental approach (*p* = 0.018).

These findings suggest that cell‐based therapies may offer potential advantages in short‐term pain control compared to standard treatments. However, additional outcomes, such as condylar volume, may serve as critical differentiators when evaluating the broader efficacy and regenerative impact of these advanced therapies.

Recently, the first global study on extensively manipulated advanced cell‐based therapy demonstrated that a single intra‐articular injection of autologous nasal septum cartilage chondroprogenitor cells was well tolerated for the treatment of mandibular condylar resorption following orthognathic surgery. This phase I/IIa clinical trial enrolled ten patients monitored over a 12‐month follow‐up period. The findings indicated that autologous chondroprogenitors effectively reduced arthralgia, improved mandibular function and either stabilised condylar volume or promoted tissue regeneration in selected cases. All patients experienced improved pain‐free and assisted mouth opening, and most condyles exhibited stabilisation of degenerative changes without significant further volumetric loss [20]. Notably, this study incorporated rigorous safety analyses, hightlighting a critical component of cell‐based therapy research.

#### 2.2.2 Emphasising Responder and Non‐Responder Profiling in Trials Design

Here, we propose a clinical trial design that extends beyond the conventional placebo‐controlled structure by profiling responders and non‐responders within the treated cohort. This approach categorises patients based on objective and subjective improvements in pain, function and imaging outcomes following regenerative therapies. In this sense, defining a non‐responder is a complex task, primarily dependent on the selected outcome measure. Responders are patients who exhibit outcomes that demonstrate the effectiveness and safety of a given treatment. Non‐responsiveness to a specific treatment may arise from various factors, including individual variability, methodological limitations, and interpretation of results. Non‐responders should be followed longitudinally to ensure safety and to monitor potential delayed effects of TMJ procedures. Additionally, non‐responders can serve as an internal control group for comparison with responders, offering insights beyond those provided by conventional placebo‐controlled designs, as previously proposed [5]. This strategy enhances data interpretation and reduces the necessity of enrolling a larger sample size [5]. Analysing non‐responders as a distinct subgroup provides valuable insights into the biological and psychosocial factors contributing to therapeutic refractoriness. Including non‐responders as an internal control facilitates comparative analyses alongside placebo or standard treatments, emphasising clinically relevant data that address the heterogeneity of TMJ‐OA. For a comprehensive evaluation of treatment efficacy and a more refined assessment of responsiveness, we recommend the following outcome measures:

##### 2.2.2.1 Quantitative Imaging

Imaging techniques should be employed to monitor changes in condylar bone shape and volume, joint space narrowing, and synovial inflammation in order to assess structural outcomes in TMJ‐OA. In this regard, both MRI and CT play a crucial role in evaluating disease progression. Studies have demonstrated that condyles presenting with surface erosion at the first clinical visit exhibit significantly reduced volume following treatment compared to those without erosion [21]. Moreover, bone resorption and deposition are more pronounced in eroded condyles with surface erosion, and the presence of joint pain is associated with a decrease in condylar volume and increased resorption. Additionally, changes in condylar volume after treatment have been shown to negatively correlate with the duration of pain relief [22]. These findings underscore the substantial impact of condylar erosion and TMJ pain on structural alterations and highlight the importance personalised treatment strategies in TMJ‐OA management consequently, achieving disease stabilisation may represent a key therapeutic goal.

##### 2.2.2.2 Pain and Function Metrics

Following the ACTTION initiative recommendations for reporting outcomes, it is essential to specify the instruments used, the frequency of measurement and the anatomical areas of pain being assessed [23]. Standardised tools, such as the Visual Analogue Scale (VAS), unassisted and assisted mandibular range of motion (ROM) measurements, and the Jaw Functional Limitation Scale (JFLS), should be employed to quantify pain intensity and functional limitations, thereby providing objective data on treatment efficacy. Additionally, quantifying the use of rescue medication can offer a more comprehensive perspective on pain management and control. Improving assay sensitivity in randomised clinical trials (RCT) may facilitate the identification of effective pain treatments. A systematic review and meta‐analysis of RCTs across various chronic pain conditions found that average pain intensity and worst pain intensity measures exhibited comparable assay sensitivity, suggesting that either measure may serve as a valid primary outcome in pain treatment trials [24]. These findings underscore the importance of selecting appropriate outcome measures to evaluate treatment efficacy and advance research in the field.

##### 2.2.2.3 Psychological and Qualitative Sensory Measures

Instruments such as the ROPA OPPERA ‐ a rapid and valid tool for stratifying patients independently of anatomical diagnosis ‐ provide valuable insights into the psychological impact of chronic orofacial pain, including that associated with TMJ‐OA, as well as the psychosocial factors influencing treatment outcomes [25]. Traditional approaches to assessing regenerative treatments outcomes often emphasise on anatomical characteristics, frequently overlooking the biopsychosocial factors that modulate pain perception and influence the progression of chronic pain [14]. By classifying patients based on psychological and sensory pain mechanisms ‐ rather than relying solely on structural outcomes ‐ these tools have the potential to enhance personalised pain assessment, particularly for individuals affected by TMJ‐OA, enabling a more comprehensive and effective evaluation of regenerative therapies.

### 2.3 Standardisation of Advanced Cell‐Based Therapy and Quality Control

Understanding the mechanisms of action underlying advanced cell‐based therapies is fundamental to defining effective therapeutic strategies. These mechanisms—such as immunomodulation, neovascularisation, fibrosis attenuation and endogenous tissue regeneration—vary depending on the cell type and tissue of origin [26]. This proposal advocates for a novel approach to clinical trials for cell‐based therapies targeting TMJ‐OA. Unlike traditional pharmaceuticals, these therapies involve the administration of cells and their derivatives, which migrate to the site of TMJ tissue injury, where their activity is modulated by the local microenvironment. This dynamic interaction necessitates a tailored approach to determining the effective dose, particularly when administering single or multiple intra‐articular injections. Such an approach allows for the identification of non‐responders through timely follow‐up assessments or surrogate assays, typically within the first 1–3 months post‐treatment, thereby optimising therapeutic monitoring and adjustment strategies.

The standardisation of cell preparation and strict adherence to regulatory requirements—such as identity, purity and potency—are critical to ensuring the reproducibility and efficacy of cell‐based therapies [27]. Regulatory frameworks mandate comprehensive characterisation of cell populations, rigorous contamination control, and potency testing to maintain therapeutic consistency. The inherent biological complexity of these therapies, including heterogeneous cell populations and ambiguous nomenclature (e.g., mesenchymal stem cells), further complicates standardisation efforts [28]. Cryopreservation techniques play a pivotal role in preserving the viability and therapeutic potential of cells, maintaining them under optimal conditions to enhance reproducibility. Combined with innovative clinical trial methodologies, these measures constitute significant advancements in the development of safe, effective and reliable clinical applications for cell‐based therapies.

## 3 Discussion

The selection of appropriate outcomes is a critical step in clinical trials involving TMJ‐OA. The American Association of Oral and Maxillofacial Surgeons (AAOMS) defines treatment success as achieving mild or no pain (VAS score ≤ 3) and a maximum mouth opening (MMO) of ≥ 35 mm at 12 months post‐treatment (AAOMS, 1984). While patients receiving saline injections often report significant pain relief lasting up to six months [29], such short‐term pain control may not consistently translate into long‐term benefits for all responders. This is particularly relevant for chronic conditions like TMJ‐OA, which may continue to progress degeneratively despite transient asymptomatic periods. Relying solely on arthralgia as the primary outcome may therefore compromise the accurate identification of long‐term responders.

Although arthralgia may be temporally associated with disc displacement [30], particularly in its early stages, this condition is frequently asymptomatic, especially in the reducing form. Disc displacement is commom among younger individuals, with the non‐reducing subtype likely representing the initial peak of intra‐articular TMJ disorder‐related disability in this population. In contrast, degenerative joint diseases are more common in older populations, characterising a second peak of TMJ‐related disability later in life [31].

Incorporating imaging‐based evidence of degenerative disease and assessing its progression is therefore essential. Although standardised guidelines for monitoring condylar degeneration via imaging are currently lacking, studies suggest that a reduction of up to 10% in condylar volume indicates stabilisation of the degenerative process [21]. Smaller reductions or increases in condylar volume can be interpreted as a positive tissue response to regenerative therapies. These structural insights provide a critical complement to symptom‐based evaluations, ensuring a more comprehensive and accurate assessment of therapeutic efficacy.

When classifying a patient as a responder, it is crucial to define the expected response intensity, determine the threshold at which a treatment response is considered achieved, and specify the duration for which the response must be sustained [32]. Accordingly, precise outcomes and time points for evaluation must be clearly established (Table 1). Furthermore, incorporating non‐responders as an internal comparison group can optimise resource allocation and reduce the required sample sizes. Adopting a responder‐based framework enhances alignment with clinical practice, where treatment is tailored to individual patient characteristics, thereby improving the applicability and clinical relevance of research findings.

It is well established that there is intrinsic variability among participants in randomised clinical trials leads to ‘response heterogeneity' ‐ clinically significant individual differences in physiological or psychological responses to the same treatment or intervention that cannot be attributed to random within‐subject variability [33] Regenerative therapies are grounded in the principles of personalised medicine, underscoring the need to evaluate individual characteristics that influence therapeutic responsiveness [34]. Beyond genetic factors, disease stage and comorbidities are critical elements that define each patient's profile, and, consequently, their response to treatment.

While conventional control groups remain essential for early‐phase safety evaluations, Phase III trials for TMJ‐OA regenerative therapies could greatly benefit from incorporating a responder/non‐responder framework. This approach shifts the focus toward clinical efficacy and individualised treatment, aiming to tailor interventions based on patient‐specific responses. Such trial designs could may also facilitate the development of predictive biomarkers for TMJ‐OA, providing clinicians with valuable tools for more precise patient selection in real‐world clinical settings. Furthermore, future studies should explore the applicability of this framework to other degenerative joint diseases, thereby contributing to the advancement of personalised medicine as a whole.

## 4 Conclusion

The responder/non‐responder clinical trial framework represents a paradigm shift with the potential to significantly enhance the precision and efficacy of TMJ‐OA treatments. By prioritising long‐term outcomes, regenerative therapies may advance toward more effective solutions for TMJ‐OA management. Acknowledging variability in treatment response enables improved patient selection and optimised therapeutic results, thereby fostering a more individualised approach to care. This proposed model underscores the need to adapt clinical trial methodologies to the multifactorial nature of TMJ‐OA, ultimately contributing to improved patient care and treatment outcomes.

## Author Contributions

Ricardo de Souza Tesch: conceptualisation, methodology, writing‐original draft, final approval. Rosana Bizon Vieira Carias: writing – review and editing; Thayanne Brasil Barbosa Calcia: conceptualisation, writing – review and editing, final approval; Priscila Grion de Miranda Borchio: writing, conceptualisation of graphical content; Esther Rieko Takamori: conceptualisation, writing – review and editing, final approval.

## Ethics Statement

The authors have nothing to report.

## Conflicts of Interest

The authors declare no conflicts of interest.

## Peer Review

The peer review history for this article is available at https://www.webofscience.com/api/gateway/wos/peer‐review/10.1111/joor.14012.

## Data Availability

Data sharing is not applicable to this article as no data sets were generated or analysed during the current study.
